# Audit, Feedback, and Education to Improve Quality and Outcomes in Transurethral Resection and Single-Instillation Intravesical Chemotherapy for Nonmuscle Invasive Bladder Cancer Treatment: Protocol for a Multicenter International Observational Study With an Embedded Cluster Randomized Trial

**DOI:** 10.2196/42254

**Published:** 2023-06-15

**Authors:** Kevin Gallagher, Nikita Bhatt, Keiran Clement, Eleanor Zimmermann, Sinan Khadhouri, Steven MacLennan, Meghana Kulkarni, Fortis Gaba, Thineskrishna Anbarasan, Aqua Asif, Alexander Light, Alexander Ng, Vinson Chan, Arjun Nathan, David Cooper, Lorna Aucott, Gautier Marcq, Jeremy Yuen-Chun Teoh, Patrick Hensley, Eilidh Duncan, Beatriz Goulao, Tim O'Brien, Matthew Nielsen, Paramananthan Mariappan, Veeru Kasivisvanathan

**Affiliations:** 1 Department of Urology, Western General Hospital Edinburgh Edinburgh United Kingdom; 2 British Urology Researchers in Surgical Training London United Kingdom; 3 Division of Surgery and Interventional Science University College London London United Kingdom; 4 Department of Urology Norfolk and Norwich University Hospital Norwich United Kingdom; 5 Department of Urology National Health Service Greater Glasgow and Clyde Glasgow United Kingdom; 6 Department of Urology University Hospitals Plymouth Plymouth United Kingdom; 7 Health Services Research Unit University of Aberdeen Aberdeen United Kingdom; 8 Department of Urology St. George's University Hospital London London United Kingdom; 9 Harvard Business School Harvard University Boston, MA United States; 10 Oxford University Hospitals Oxford United Kingdom; 11 Department of Surgery and Cancer Imperial College London London United Kingdom; 12 Urology Department Claude Huriez Hospital Centre Hospitalier Universitaire de Lille Lille France; 13 Cancer Heterogeneity Plasticity and Resistance to Therapies Institute Pasteur de Lille University of Lille Lille France; 14 S H Ho Urology Centre Department of Surgery The Chinese University of Hong Kong Hong Kong Hong Kong; 15 Department of Urology College of Medicine University of Kentucky Lexington, KY United States; 16 Department of Urology Guy's and St. Thomas' National Health Service Foundation Trust London United Kingdom; 17 Department of Urology University of North Carolina Medical School Chapel Hill, NC United States; 18 Edinburgh Bladder Cancer Surgery Department of Urology Western General Hospital Edinburgh Edinburgh United Kingdom

**Keywords:** TURBT, bladder cancer, quality improvement, performance feedback, transurethral resection, urology, oncology, recurrence, surgery, surgical, quality indicator, performance, feedback, evaluation

## Abstract

**Background:**

Nonmuscle invasive bladder cancer (NMIBC) accounts for 75% of bladder cancers. It is common and costly. Cost and detriment to patient outcomes and quality of life are driven by high recurrence rates and the need for regular invasive surveillance and repeat treatments. There is evidence that the quality of the initial surgical procedure (transurethral resection of bladder tumor [TURBT]) and administration of postoperative bladder chemotherapy significantly reduce cancer recurrence rates and improve outcomes (cancer progression and mortality). There is surgeon-reported evidence that TURBT practice varies significantly across surgeons and sites. There is limited evidence from clinical trials of intravesical chemotherapy that NMIBC recurrence rate varies significantly between sites and that this cannot be accounted for by differences in patient, tumor, or adjuvant treatment factors, suggesting that how the surgery is performed may be a reason for the variation.

**Objective:**

This study primarily aims to determine if feedback on and education about surgical quality indicators can improve performance and secondarily if this can reduce cancer recurrence rates. Planned secondary analyses aim to determine what surgeon, operative, perioperative, institutional, and patient factors are associated with better achievement of TURBT quality indicators and NMIBC recurrence rates.

**Methods:**

This is an observational, international, multicenter study with an embedded cluster randomized trial of audit, feedback, and education. Sites will be included if they perform TURBT for NMIBC. The study has four phases: (1) site registration and usual practice survey; (2) retrospective audit; (3) randomization to audit, feedback, and education intervention or to no intervention; and (4) prospective audit. Local and national ethical and institutional approvals or exemptions will be obtained at each participating site.

**Results:**

The study has 4 coprimary outcomes, which are 4 evidence-based TURBT quality indicators: a surgical performance factor (detrusor muscle resection); an adjuvant treatment factor (intravesical chemotherapy administration); and 2 documentation factors (resection completeness and tumor features). A key secondary outcome is the early cancer recurrence rate. The intervention is a web-based surgical performance feedback dashboard with educational and practical resources for TURBT quality improvement. It will include anonymous site and surgeon-level peer comparison, a performance summary, and targets. The coprimary outcomes will be analyzed at the site level while recurrence rate will be analyzed at the patient level. The study was funded in October 2020 and began data collection in April 2021. As of January 2023, there were 220 hospitals participating and over 15,000 patient records. Projected data collection end date is June 30, 2023.

**Conclusions:**

This study aims to use a distributed collaborative model to deliver a site-level web-based performance feedback intervention to improve the quality of endoscopic bladder cancer surgery. The study is funded and projects to complete data collection in June 2023.

**Trial Registration:**

ClinicalTrials.org NCT05154084; https://clinicaltrials.gov/ct2/show/NCT05154084

**International Registered Report Identifier (IRRID):**

DERR1-10.2196/42254

## Introduction

### Background

Nonmuscle invasive bladder cancer (NMIBC) is a common disease with an incidence of 19.1 and 4.0 cases (men and women, respectively) per 100,000 person-years in the European Union [1]. Three-quarters of all new bladder cancer diagnoses are that of NMIBC [2]. This disease is the most expensive to manage, per patient, from diagnosis to death [3,4].

**Figure 1:**
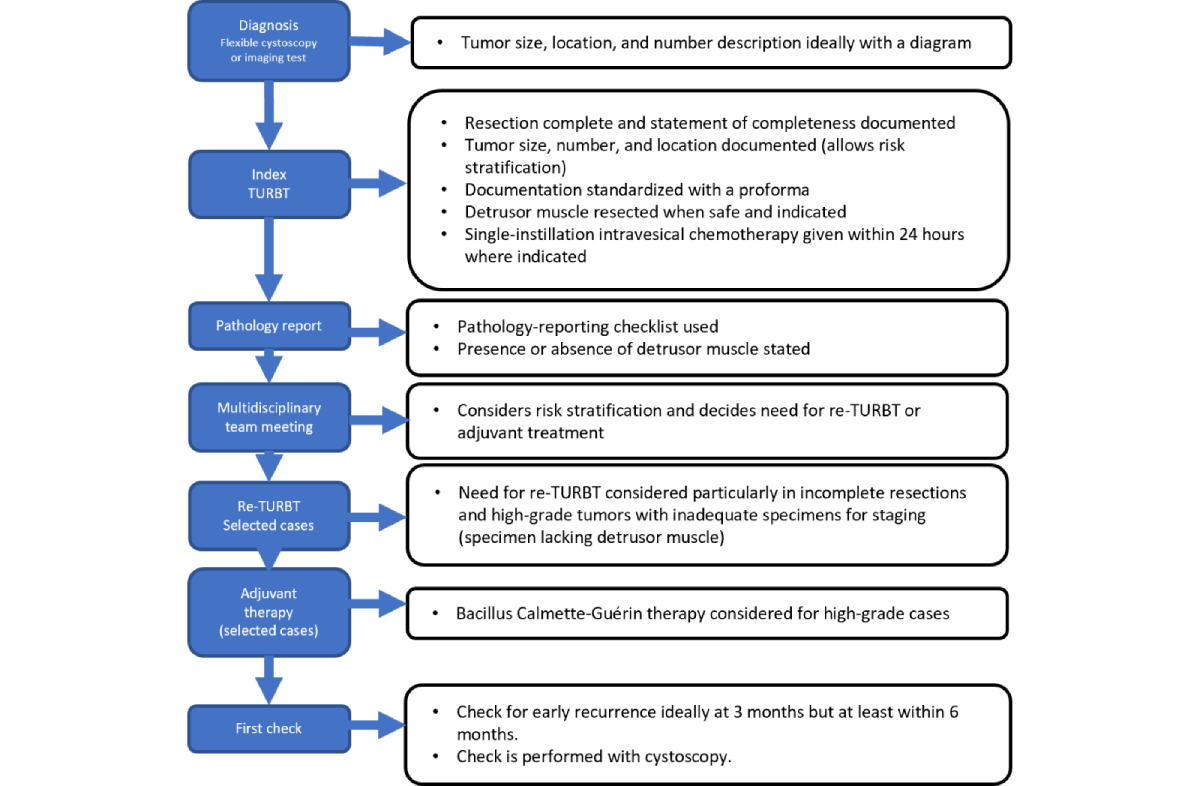
Nonmuscle invasive bladder cancer diagnosis and treatment pathway. TURBT: transurethral resection of bladder tumor.

The standard of care management for all suspected NMIBC is surgical transurethral resection of bladder tumor (TURBT). Evidence guides how the initial TURBT should be performed and perioperative practice, including the use of single-instillation intravesical chemotherapy (SI-IVC) to reduce recurrence rates of cancer [5]. It has been proposed that achieving “good-quality” NMIBC surgery and perioperative practice results in reduced recurrence rates [6]. This evidence forms the basis for the recommendations made in guidelines including those by the European Association of Urology (EAU) [5], the American Urological Association (AUA) [7], and the National Institute for Health and Care Excellence (NICE) [8] in England and Wales. The usual NMIBC diagnosis and treatment pathway is shown in Figure 1.

### The Problem

Compliance with best practice for TURBT surgery and perioperative guidelines varies widely [9], which may impact oncological outcomes [10-12]. There is a significant variation in early tumor recurrence rates between different sites that cannot be accounted for by patient, tumor, or adjuvant treatment factors [10], suggesting surgical factors may be the cause. In a survey of practice in 9 European countries published in 2019, significant deviation from guidelines was identified even for interventions with high certainty of evidence, such as SI-IVC administration [9]. There has recently been a proposed set of “quality indicators” for TURBT but the published review acknowledged that there was a lack of evidence that implementing these quality indicators resulted in improved outcomes [13]. A long-running study on quality standards in TURBT surgery in Scotland demonstrated that being involved in a national audit and feedback program of TURBT quality performance indicators did lead to a trend in improvement of quality indicator achievement over time, but a critical limitation was that there was no control group or comparison to baseline performance [14,15]. This work also established the utility and measurability of certain TURBT quality indicators in the real world.

### The Intervention: Electronic Audit, Feedback, and Education

Audit and feedback have the potential to improve professional practice and impact patient outcomes. Meta-analyses of the effects of audit and feedback in health care demonstrate wide variation in effect size [16]. The median baseline performance–adjusted improvement in compliance with desired practice was 4.3% but varied from 0.5% to 16%. Further, there is theory and evidence that education on addressing deficiencies highlighted by audit may enhance the effectiveness of audit and feedback [17,18].

A systematic review concluded that feedback can improve surgical performance, but the strength of the conclusions was tempered by the heterogeneity, the lack of randomization, and the lack of controls in the included studies [15,19,20]. A systematic review of electronic audit and feedback interventions concluded that effects were variable and only 2 of 7 identified studies used theory to guide interventions [21].

For bladder cancer surgery, there are professional practice behaviors under the control of the surgeon that have proven to be associated with patient outcomes after TURBT surgery [6,22-25]. Given that audit, feedback, and education is a cost-effective and simple intervention and has demonstrated efficacy in other settings, we would hypothesize that this intervention also improves perioperative indicators of surgical quality. Furthermore, there is evidence that targeting “process measures” as the unit for change is more effective than targeting patient outcomes [26]. Therefore, we chose to target professional practice behaviors (or quality indicators) as the primary outcomes rather than a disease outcome such as recurrence rates.

### Objectives

#### Main Objective

The primary objective is to determine whether “audit, feedback, and education” improves achievement of TURBT quality indicators.

#### Other Objectives

The other objectives are to determine (1) if audit, feedback, and education improves the early recurrence rate of NMIBC after first TURBT; (2) if achievement of TURBT quality indicators is associated with early recurrence rate of NMIBC after the first TURBT; (3) the average and variation in achievement of TURBT surgery quality indicators and early recurrence rate across surgeons, sites, countries, and health care settings (site type and operating list type); and (4) associations among site, surgeon, and surgical factors and achievement of TURBT quality indicators and early recurrence rate.

## Methods

### Study Design

This is an international, observational, multicenter study with an embedded prospective cluster–cluster randomized, parallel-arm, superiority trial of audit, feedback, and education. A cluster randomized trial was chosen because intrasite contamination would be expected if randomization was at the clinician level. A cluster is defined as one or more hospitals within the same health care organization where the same surgeons perform TURBT surgery. Sites will be informed of any updates to the protocol or standard operating procedures with the latest trial documents available on the BURST RESECT study website (protocol version 4.0 November 11, 2020).

### Data Collection, Management, and Privacy

Data will be entered into a web-based secure database hosted at University College London (Research Electronic Data Capture [REDCap]). Collected data are anonymous. Case report forms were developed with peer review and national and international testing (tested in the United Kingdom, France, and the United States). Data range checks, data sense rules, and essential required variable alerts will be programed at outset to improve accuracy and completeness. In total, 10% of all records will be manually checked for sense and completeness. Manually identified errors will be coded as automated rules, and the whole database scanned or audited for errors. Identified errors will be alerted to sites for correction before database lock. Data errors and incomplete or missing follow-up data will be queried with sites on a patient-by-patient basis through a web-based data auditing app. The resection and single-instillation intravesical chemotherapy for nonmuscle invasive bladder cancer treatment study data analysis group comprising statisticians based at the University of Aberdeen will have access to the final data set.

### Ethical Considerations

Local and national ethical and institutional approval or exemption will be obtained at each participating site. In the United Kingdom, the University of Aberdeen deemed this study exempt from research ethical approval in line with health research authority guidelines. There is no compensation to sites associated with this study. The study will be published in peer-reviewed journals and presented at national and international scientific congresses. Individual sites or surgeons will not be identified in study reports or performance feedback.

### Inclusion and Exclusion Criteria

#### Site Inclusion and Exclusion Criteria

Sites anywhere in the world will be included if they perform TURBT for NMIBC and could include at least 20 consecutive eligible cases in a period of 12 months. Sites will be excluded if they fail to include 20 cases with a minimum required data set to determine baseline performance. Where a site requests removal from the study, data collected to date will be maintained for reporting purposes unless the site requests deletion.

#### TURBT Case Inclusion and Exclusion Criteria

Sites will include consecutive cases of first, curative intent, elective transurethral surgery for a new diagnosis of urothelial carcinoma, thought to be nonmuscle invasive at the time of surgery, across all surgeons at their site. Cases with known concurrent upper tract urothelial carcinoma will be excluded.

### Study Design

**Figure 2:**
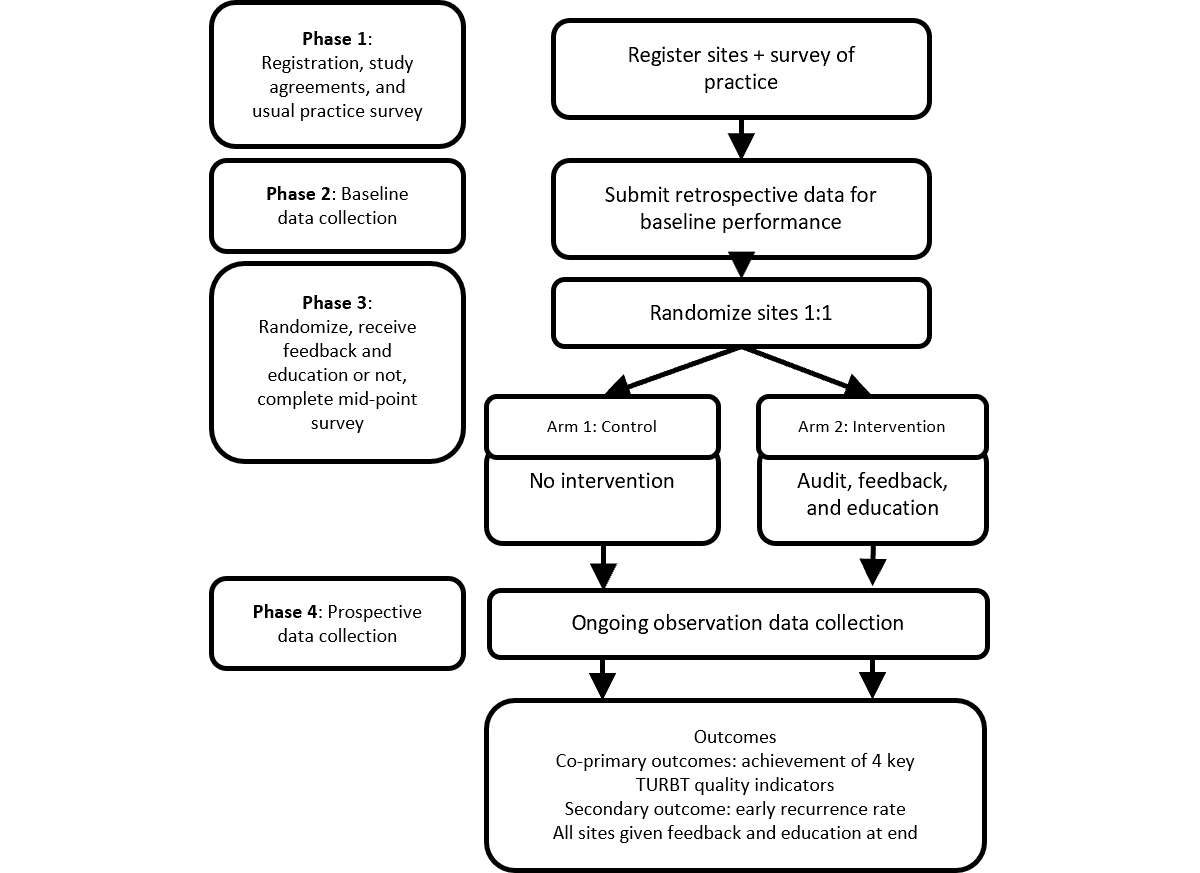
Study phases and design. TURBT: transurethral resection of bladder tumor.

The study will have four phases, which are summarized in Figure 2: (1) phase 1: site registration, study agreements, and usual practice surveys; (2) phase 2: retrospective baseline data collection; (3) phase 3: randomization to either arm of “no intervention,” or “audit, feedback, and education”; and (4) phase 4: prospective data collection.

#### Site Registration, Study Agreements, Surgeon Registration, and Usual Practice Surveys

Sites will complete a survey about their location, hospital type, and their usual practice. An agreement will be signed by the site principal investigator including consent to randomization. Each surgeon performing TURBT at the site will be registered along with details about their surgical experience including their grade, number of TURBTs performed per month, and years of experience.

#### Baseline Data Collection

Sites will be asked to enter data about consecutive eligible TURBT cases performed at their site counting backward from the date of site registration. The rationale is to sample cases from before a site had awareness of the study to obtain a cohort free from the Hawthorne effect [27].

#### Randomization

Sites will be randomized 1:1 in parallel arms when the submission of the minimum required retrospective data is complete. Sites will be randomized to either (1) no intervention (the site continues to submit data on consecutive eligible cases but without performance feedback and education during the study). Once the study is complete, these sites will be provided with performance and education feedback or (2) audit, feedback, and education (performance feedback and education provided before beginning further prospective phase of study).

Sites will be randomized using the randomization app at the Centre for Healthcare Randomised Trials (CHaRT), University of Aberdeen, which developed the randomization sequence. This randomization app is available 24 hours a day, 7 days a week as a web-based app. Randomization will be minimized for site-level variables that are thought to significantly impact achievement of TURBT quality indicators. There is no blocking. These are baseline performance from the baseline audit, geographical region (continent, except that the United Kingdom is considered alone, Australia will be combined with mainland Europe), and the presence of an existing TURBT audit at that site before the study. In addition, a random component is used in the minimization algorithm to ensure concealment of the allocation.

The study administrator will access the web-based system. They will enter the minimization variables into the web-based system, which returns the allocation status. The chief investigator of the site will be informed of their allocation following randomization. There is no blinding. To prevent contamination following randomization, sites randomized to no intervention will not be granted access to the dedicated personalized feedback and education resources, which are username and password protected.

#### Data Management

Local data will be routinely collected health care data accessed by members of the clinical team. Nonidentifiable data will be manually entered into the secure, password-protected study database (hosted at University College London) after appropriate local approvals have been obtained. Only the study management and statistical team will have access to the full data set.

## Results

### Audit, Feedback, and Education Design

The audit, feedback, and education intervention was designed using evidence-based theories about interventions that cause behavior change. These theories were both general [16,18,28-31] and specific to TURBT surgery based on our previous work [12,32].

The intervention is delivered at the site level with recommendations about how to engage with, disseminate, and use the feedback and education. There is no mandated change to clinician behavior or patient care, all patient treatment and professional practice decisions are at the discretion of the clinician. Sites can request to leave the study at any time, and all withdrawals will be reported. It will not be permitted for a site allocated to “no intervention” to receive the intervention during the study period.

Our group previously used qualitative methods to assess barriers and facilitators to a coprimary outcome for this study: SI-IVC administration [32]. The data from this qualitative study and a systematic review of barriers to NMIBC guideline compliance [12] were used to identify intervention functions and behavior change techniques (BCTs) using the theoretical domains framework, as suggested in the implementation science literature [29,30,33].

Conclusions from systematic reviews and expert groups about improving effectiveness of audit and feedback were used to inform the design of the feedback and education. For example: “feedback may be more effective when baseline performance is low [16]”; “if the source is a supervisor or colleague [16]”; “it is delivered in both verbal and written formats [16]”; “it includes both explicit targets and an action plan [16]”; and “process of care measures are more positively influenced by feedback than outcome of care [26].” In addition, adding education to feedback may enhance the effectiveness of audit and feedback and so this strategy will be adopted [17]: “audit and feedback interventions will be more effective when recommendations related to the audit and feedback are based on good quality evidence” [18] and if it “creates opportunity to learn [18].”

The design of the feedback is further informed by Brehaut et al’s [34] “15 suggestions for optimising effectiveness” of practice feedback interventions, the Clinical Performance Feedback Intervention Theory [31], and Colquhoun et al’s [18] “Theory Informed Hypotheses” for improving effectiveness of audit and feedback.

Finally, the study steering group applied the APEASE (Affordability, Practicability, Effectiveness and Cost-effectiveness, Affordability, Side-effects, Equity) [29] criteria to the possible BCTs. This process highlighted suitable BCTs and feedback and education components detailed in Table 1.

**Figure 3:**
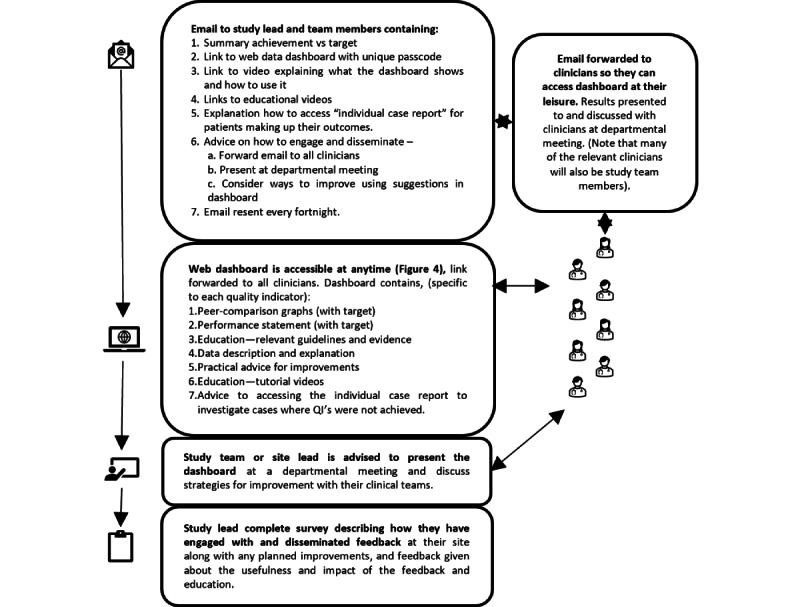
Intervention and mode of delivery. QI: quality indicator.

**Figure 4:**
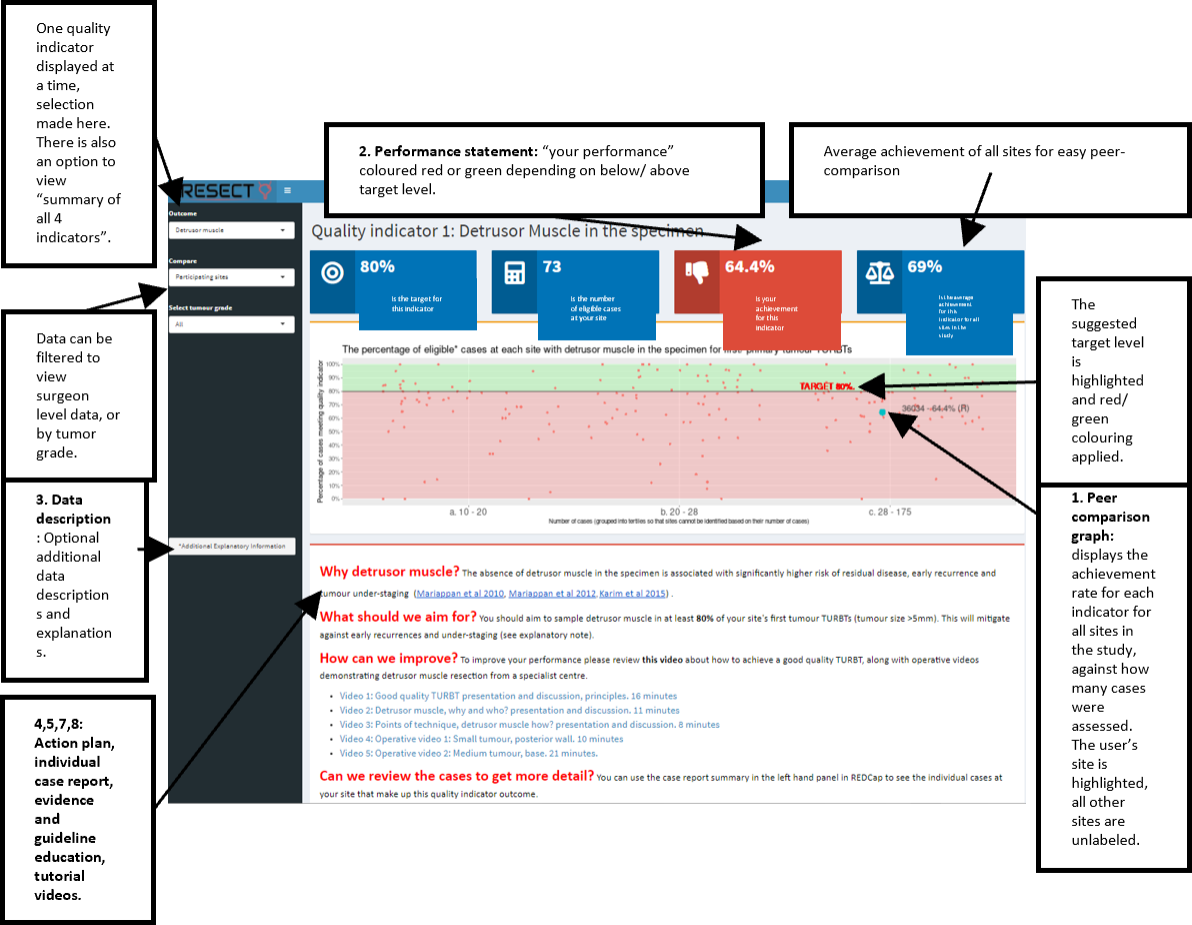
Intervention and design. Web-based performance feedback and education dashboard—accessible to only sites in the intervention arm during the study. Numbers in the boxes relate to the components in Table 1.

The performance feedback will take the form of a web-based feedback dashboard that will be freely available at any time to those given access as well as a report of the individual cases that make up their performance outcomes and other components. The components of the feedback dashboard are described in Table 1 and Figure 3 and displayed in Figure 4.

The study team or lead investigator will receive an email detailing the feedback and education intervention. They will be asked to forward the email and feedback and education dashboard access to all the surgeons at their site and present and discuss the feedback at a departmental meeting. The lead investigator must then complete a survey about how the feedback and education were used and disseminated at their site and any specific changes they have made (Figure 3).

**Table 1:** Feedback and education intervention components, behavioral change techniques, theories, and mode of delivery.

Component		Description	Behavior change technique [30] or audit and feedback effectiveness theories or evidence	Mode of delivery
**Performance feedback**				
	1. Peer-comparison graphs	“Rate of achievement” scatter graphs displayed at both the site and surgeon level. All sites and surgeons are displayed but are anonymous, only current site data points are labeled with anonymous unique ID. Filterable by tumor grade.	Social comparison (6.2), “for those with a mastery goal orientation” feedback will be more effective if it involves comparison to others [18].	Web-based data dashboard, accessed via link in feedback email with site-specific passcode.
	2. Performance statement	The site’s level of achievement, the average level of achievement, the target, and whether they are considered above or below an explicit target. This is based on the experience of the Scottish Quality Performance Indicator programme [15].	Social comparison (6.2), Explicit target given [16].	In feedback email and displayed alongside peer-comparison graphs on web dashboard.
	3. Data description	An optional pop-up box described how the graphs were generated including the description of numerator and denominator, advice on interpretation, justification of quality indicator selection, target and case selection.	Theory of trust or credibility and nature of the data [18].	An optional pop-up box alongside each peer-comparison graph in the web dashboard.
	4. Action plan	Actions specific to each behavior provided, for example, an operative recording proforma to improve documentation outcomes.	Instruction on how to perform the behavior (4.1), restructuring the physical environment (12.1), adding objects to the environment (12.5), prompts or cues (7.1).	Displayed beneath the peer-comparison graph of each quality indicator with links to either written resources or to download the operative proforma.
	5. Individual case report	The cases analyzed for each quality indicator, along with selected clinically relevant variables (eg, tumor details and operating surgeon code) can be reviewed in a filterable report, and individual database records easily entered for case review and discussion.	Theory of feedback specificity, user-guided experience, and social engagement [18].	The site-specific reports are available in the study REDCap^a^ database environment, instructions about how to use are included in the feedback email and dashboard.
	6. Provide feedback more than once	The feedback dashboard is available throughout and displays live performance. Sites will be encouraged to engage with this at a departmental level regularly. Feedback email is resent encouraging ongoing feedback engagement every 2 weeks.	Provide multiple instances of feedback [34].	Same delivery method.
**Education**				
	7. Evidence and guideline education	Key evidence and related guidelines about why the behavior is important are summarized and links are provided to published evidence.	Information about health (5.1) and social and environmental consequences (5.2).	Provided beneath the peer-comparison graph for each of the quality indicators. Links to web-hosted publicly available resources. Accessible at any time after dashboard access is given.
	8. Tutorial videos	Evidence reviewed and practice from a center of excellence presented by an expert in the field. Operative video tutorials for practical skills provided. The videos can be accessed at any time, and access is unsupervised. Collaborators are encouraged to review them together at departmental meeting but they may also be accessed individually.	Instruction on how to perform the behavior (4.1) and demonstration on how to perform the behavior (6.1) by a credible source (9.1).	Links to web-hosted videos provided beneath the peer-comparison graphs on the dashboard and in the feedback email. Accessible at any time after dashboard access is given.

^a^REDCap: Research Electronic Data Capture.

### Prospective Data Collection After Randomization

Sites will be asked to submit observational data about all consecutive eligible TURBT cases performed after they have been randomized. Sites in the feedback and education arm will only be permitted to submit cases in the prospective phase once the mid-point survey has been received. The purpose of the midpoint survey was to determine if any unprompted changes to practice or performance had been made since the site registration survey was completed and confirm that sites had disseminated the education and feedback at their site (if relevant) and assess how this had been done.

### Primary Outcomes

#### Principles

The study will use 4 coprimary outcomes. The primary outcomes are key “surgical quality indicators” or “process measures.” The outcomes were chosen through a combination of evidence, guidelines associating their performance with improved bladder cancer outcomes, expert steering group consensus, and independent external peer review. Primary outcomes were chosen to meet the following criteria: (1) They are behaviors or practices associated with the primary operation or occurring at the time of the primary operation. (2) It is possible for the surgeon to directly influence these practices. (3) They are practices recommended by professional guidelines or evidence associating them with improved outcomes. (4) They can be objectively measured through review of the standard patient record.

Coprimary outcomes were chosen because not all behaviors are applicable to all patients and no single behavior may indicate overall quality. The 4 outcomes chosen draw on experience and the outcomes used in the Scottish Quality Performance Indicator programme and work by Mariappan et al [14,15].

The intervention will be considered effective if any one of the 4 coprimary outcomes is significantly improved. “Eligible cases” and excluded cases were determined for each coprimary outcome. Eligible cases (after exclusions) make up the denominator for calculation of the proportion of cases achieving the outcome.

#### Quality Performance Indicator 1: The Proportion of Eligible Cases Where Detrusor Muscle Is Present in the Surgical Specimen

##### Rationale

International evidence-based guidelines include clear recommendations related to TURBT [5,7]. There is evidence that resection of detrusor muscle at the index TURBT is associated with reduced early recurrence rate [6,22] and long-term mortality [25] and is more likely to be resected by experienced than inexperienced surgeons [6,22]. If detrusor muscle is not obtained, it is not possible to accurately determine the tumor stage and all of the tumor may not have been removed, which may lead to recurrence. It is accepted that detrusor muscle resection is not appropriate in all patients and is a clinical judgment. Therefore, the average rate of detrusor muscle will be used as a performance indicator.

##### Eligibility

All cases where the tumor maximal diameter is estimated at >5 mm by the operating surgeon.

##### Assessment

The outcome will be determined from the surgical pathology report.

##### Exclusions

Where the data for the presence or absence of detrusor muscle in the specimen are investigator-missing, the case will be excluded.

#### Quality Performance Indicator 2: The Proportion of Eligible Cases Where SI-IVC Is Administered Within 24 Hours of TURBT

##### Rationale

There is high-certainty evidence supporting the use of SI-IVC, such as Mitomycin C following TURBT. It is known that SI-IVC administration can significantly reduce the rate of NMIBC recurrence [23,35-39]. EAU and AUA guidelines recommend administration of SI-IVC within 24 hours of surgery in patients most likely to benefit [5,7].

##### Eligibility

All cases at sites where it is possible to give SI-IVC.

##### Assessment

The outcome will be determined from review of the patient record.

##### Exclusions

It is anticipated that a small number of sites will be unable to provide SI-IVC due to local or national service, supply, or economic restraints. These sites will be identified from the study registration survey and excluded from this indicator a priori. Cases where a patient has a documented allergy to SI-IVC will be excluded. Where this data field is investigator-missing, the case will be excluded from this outcome.

#### Quality Performance Indicator 3: The Proportion of Eligible Cases Where Resection Completeness Is Documented in the Operation Record

##### Rationale

Guidelines strongly recommend documenting the completeness of resection [5]. Using a TURBT proforma to document the surgical procedure and findings at surgery in a standardized way is associated with improved patient outcomes [15,24,40]. Completely removing all visible tumors is a core surgical principle in cancer surgery. Documentation of this is essential for subsequent multidisciplinary team meeting decisions on the management of individual patients as it determines the need for repeat resection, which can improve oncological outcomes in selected patients [41].

##### Eligibility

All cases included in the study.

##### Assessment

The outcome will be determined from review of the operation note. To achieve the indicator, a statement that the resection was either complete, incomplete, or uncertain is required. Where there is no statement of resection completeness in the operation record the indicator will be failed.

##### Exclusions

Where this data field is investigator-missing, the case will be excluded from this outcome.

#### Quality Performance Indicator 4: The Proportion of Eligible Cases Where All of the Tumor Number, Size, and Location Are Documented in the Operation Record

##### Rationale

Guidelines strongly recommend documenting the tumor location, appearance, size, and multifocality [5]. Tumor size and number are associated with recurrence risk and are required to risk-stratify patients according to European Organisation for Research and Treatment of Cancer (EORTC) risk categories as recommended by both AUA and EAU guidelines [5,7]. Documentation of these factors and subsequent risk stratification allows multidisciplinary team meeting decisions for individual patients, determining the need for adjuvant treatments, which determine long-term oncological outcomes. Documenting tumor location also allows appropriate assessment of recurrence and resection completeness at reresection surgeries and during cystoscopic surveillance.

##### Eligibility

All cases included in the study.

##### Assessment

The outcome will be determined from review of the official operation note. To achieve the indicator, a statement of the tumor number, size (an assessment of tumor maximal diameter and at least to a resolution of <3 cm or ≥ 3 cm [a risk category cut-off in the EAU guidelines]), and location is required. Where record of any of these 3 features is missing from the operation record, the outcome will be failed.

##### Exclusions

Where this data field is investigator-missing, the case will be excluded from this outcome.

### Secondary Outcomes

#### Key Secondary Outcome: Early Recurrence Rate

##### Rationale

International guidelines recommend that a “check” cystoscopy be performed approximately 3 months after complete TURBT [5,7]. The rationale is that recurrence detected at this early stage is due to surgical failure rather than disease process [6]. Therefore, the early recurrence rate represents an outcome of surgical quality.

##### Eligibility

All cases where a cystoscopy has been performed within 6 months of complete index TURBT or planned completion re-TURBT, or, in cases where an adjuvant treatment course is given prior to check cystoscopy, at a check cystoscopy performed within 12 months.

##### Assessment

The outcome will be determined from review of the patient record. Recurrence will be defined as either (1) a clinical assessment of “highly likely” recurrence where fulguration is applied, and no surgical specimen is obtained or (2) when a surgical specimen is obtained, and then histological confirmation of cancer will be required.

##### Exclusions

Where this data field is investigator-missing. Where muscle-invasive bladder cancer is identified in either the primary or re-TURBT pathology. Where the patient is deemed not fit or appropriate for further surveillance.

#### Other Secondary Outcomes

Other secondary outcomes include (1) the proportion of eligible patients in whom all applicable primary quality indicators are achieved (not all quality indicators are applicable in every patient) and the association of this composite outcome with early recurrence rate and (2) the rate of complications of grade 3 or greater, within 30 days of surgery according to the Clavien-Dindo [42] classification.

### Sample Size

The intervention will be delivered to centers, and so the primary outcomes will also be measured at the center level. The only published data about intersite achievement of the listed coprimary outcomes are the Scottish Quality Performance Indicator programme covering 11 sites in Scotland. The outcome with the largest SD (SI-IVC) was used to determine the overall sample size. With mean 0.651 (SD 0.2) for SI-IVC, recruiting 172 sites (86 per arm) would give 90% power to detect a difference of 0.1 between the intervention and control groups at the 5% significance level. Where a smaller SD of 0.1 (detrusor muscle in specimen) or 0.15 (resection documenting) can be assumed, this would provide 90% power at the 5% significance level to detect differences of 0.05 and 0.075, respectively. It is possible that some sites may have missing data for 1 or more of the coprimary outcomes. Missing data will not be imputed for the primary outcomes. In that case, the site will be excluded from the analysis for that outcome. A sensitivity analysis will be performed to determine the effect of missing data in that outcome. An extensive international marketing campaign will be undertaken to ensure sample size is reached, along with endorsement of the study by the British Association of Urological Surgeons to all urology centers in the United Kingdom.

### Analysis

The average rate of achievement of each of the 4 co-primary outcomes per site will be compared between sites that received education and feedback and those that did not. The primary outcomes will be analyzed using mixed effects linear regression to compare the outcome between the group that received audit, feedback, and education and the control group. The primary outcomes are the proportion of cases where the relevant outcome is obtained and measured at the site level (a continuous variable). The regression model will have fixed effects for the study arm (intervention group); audit already in place at the site before the study; and the baseline achievement of the relevant outcome. A random effect will be included for the region. Outcomes will be assessed in subgroups of low- and high-grade tumors, tumors with estimated maximum diameter of ≤3 cm or >3 cm and in single vs multiple tumors for the detrusor muscle, and intravesical chemotherapy outcomes.

The early recurrence rate is a secondary outcome. This is measured at the patient level. In this instance, the outcome is binary. A mixed effect logistic regression will be used including random effects for site and surgeon to incorporate the 2 levels of clustering. Fixed effects will be included for intervention arm and tumor variables known to be associated with early recurrence (tumor size, tumor number, grade, and stage). Patient age will also be included as a fixed effect. A second analysis will be undertaken for the early recurrence outcome at the patient level to determine if the quality indicators are associated with reduced recurrence rate regardless of performance feedback.

### Secondary Analyses

This study is an observational audit with an embedded cluster randomized design and was planned so that the following factors could be analyzed for association with TURBT quality indicator achievement and early recurrence rate: (1) surgeon factors: (a) the experience of the operating surgeon in years, (b) total number of cases performed by the surgeon to date, (c) number of cases performed per month on average in the last year, and (d) a self-declared specialist interest in bladder cancer; (2) TURBT surgery performed on a dedicated TURBT list; (3) the estimated annual TURBT case volume of the site; (4) the use of en-bloc resection; (5) the use of diagnostic visual aids including: (a) photodynamic diagnosis–assisted resection, (b) narrow band imaging, and (c) Storz Professional Image Enhancement System; (6) the length of inpatient stay; (7) geographical region—continent; (8) World Health Organization health care quality and access index for the country; (9) the preexistence of a TURBT audit at the site; (10) the use of re-TURBT; (11) the use of primary adjuvant BCG or Mitomycin C; and (12) patient factors: age and sex.

## Discussion

This study aims to use a distributed collaborative model to deliver a site-level web-based performance feedback intervention to improve the quality of endoscopic bladder cancer surgery. The goal is to gain incremental benefit across vast numbers of patients using a simple and cheap intervention that will amount to large absolute benefits across the population of patients with NMIBC. We have considered previous advice from systematic reviews of previous audit and feedback interventions and used the theoretical domains framework to design the feedback intervention. We have worked closely with expert international clinical, methodological, and statistical professionals to ensure quality of study design.

The effectiveness of audit and feedback in improving professional practice varies depending on “baseline performance and how feedback is provided” as well as setting [16]. We have determined that a clinically relevant relative difference between intervention and control arms is 10% and powered this study accordingly. Electronic audit and feedback have also been found to have variable effectiveness, and a systematic review recommended closer attention to theoretical domains targeted [21]. We have done this as part of the study design. This study is unique because of its global multicenter reach and therefore aspires to power a cluster-cluster randomized controlled study design to confidently determine the effect of the intervention. The Hawthorne effect will be accounted for since we measure performance retrospectively (when the study was not known about) and then compare 2 parallel arms in the randomized study. There have been previous time-series studies of audit and feedback in TURBT surgery, which suffer from the lack of a contemporaneous control group [15,40]. To our knowledge, this will be the first randomized study of audit and feedback in TURBT surgery. Limitations of the study design include the pragmatic intervention—clinicians can choose to engage or not, and implementation at the site level is left up to local teams. The site-level intervention and analysis will limit the ability to make conclusions about the behavior of individuals. In conclusion, this study will determine if audit, feedback, and education is effective in improving professional practice in the quality of TURBT surgery and if this reduces NMIBC early recurrence rates.

## Abbreviations

APEASE: Affordability, Practicability, Effectiveness and Cost-effectiveness, Affordability, Side-effects, Equity
AUA: American Urological Association
BCT: behavior change technique
CHaRT: Centre for Healthcare Randomised Trials
EAU: European Association of Urology
EORTC: European Organisation for Research and Treatment of Cancer
NICE: National Institute for Health and Care Excellence
NMIBC: nonmuscle invasive bladder cancer
REDCap: Research Electronic Data Capture
SI-IVC: single-instillation intravesical chemotherapy
TURBT: transurethral resection of bladder tumor

## Data Availability

The data sets generated and analyzed during this study are not publicly available due to a need to maintain anonymity of the included sites as set out in the study agreement but are available from the corresponding author on reasonable request. All statistical code will be made available upon request.
